# Interaction between nitrate and trichloroethene bioreduction in mixed anaerobic cultures

**DOI:** 10.3389/fmicb.2024.1504235

**Published:** 2025-01-15

**Authors:** Dong-Mei Yang, Fen-Li Min, Ying Li, Jia-Lu Ling, Hui-Xian Zhong, Yu-Chun Xia, Ying Feng, Li-Ya Zhao, Zhao-Hua Li, Li-Lian Wen

**Affiliations:** ^1^College of Resource and Environmental Science, Hubei University, Wuhan, China; ^2^Hubei Key Laboratory of Environmental and Health Effects of Persistent Toxic Substances, School of Environment and Health, Jianghan University, Wuhan, China

**Keywords:** trichloroethene, nitrate, reductive dechlorination, self-alkalization, pH control

## Abstract

Bioremediation of trichloroethene (TCE)-contaminated sites often leads to groundwater acidification, while nitrate-polluted sites tend to generate alkalization. TCE and nitrate often coexist at contaminated sites; however, the pH variation caused by nitrate self-alkalization and TCE self-acidification and how these processes affect nitrate reduction and reductive dichlorination, have not been studied. This study investigated the interaction between nitrate and TCE, two common groundwater co-contaminants, during bioreduction in serum bottles containing synthetic mineral salt media and microbial consortia. Our results showed that TCE concentrations up to 0.3 mM stimulated nitrate reduction, while the effect of nitrate on TCE reductive dechlorination was more complex. Nitrate primarily inhibited the reduction of TCE to dichloroethene (DCE) but enhanced the reduction of vinyl chloride (VC) to ethene. Mechanistic analysis suggested that this inhibition was due to the thermodynamic favorability of nitrate reduction over TCE reduction, while the promotion of VC reduction was linked to pH stabilization via self-alkalization. As the initial nitrate concentration increased from 0 to 3 mM, the relative abundance of putatively denitrifying genera, such as *Petrimonas* and *Trichlorobacter*, increased. However, the abundance of fermentative *Clostridium* sharply declined from 31.11 to 1.51%, indicating strong nitrate inhibition. Additionally, the relative abundance of *Dehalococcoides*, a genus capable of reducing TCE to ethene, slightly increased from 23.91 to 24.26% at nitrate concentrations up to 0.3 mM but decreased to 18.65% as the nitrate concentration increased to 3 mM, suggesting that *Dehalococcoides* exhibits a degree of tolerance to high nitrate concentrations under specific conditions. Overall, our findings highlight the potential for simultaneous reduction of TCE and nitrate, even at elevated concentrations, facilitated by self-regulating pH control in anaerobic mixed dechlorinating consortia. This study provides novel insights into bioremediation strategies for addressing co-contaminated sites.

## 1 Introduction

Trichloroethene (TCE) is commonly associated with various human activities, including its use as a metal detergent, dry cleaning agent, pesticide solvent, and chemical feedstock (DRC, 2012; Ottosen et al., 2020). TCE, along with its breakdown products, dichloroethene (DCE) and vinyl chloride (VC), are known to be teratogenic, carcinogenic, and mutagenic, posing serious threats to human health (Ordaz et al., 2017). Microbial anaerobic reductive dechlorination is an effective method for eliminating TCE from water systems. This process sequentially converts TCE to DCE, then to VC, and finally to non-toxic ethene. Many dechlorinating bacteria, including species from Proteobacteria (such as *Geobacter*, *Desulfomonile*, and *Desulfuromonas*) and Firmicutes (such as *Dehalobacter* and *Desulfitobacterium*), can partially catalyze the reduction of TCE to DCE, while *Dehalococcoides mccartyi* and *Dehalogenimonas*, which belong to the phylum Chloroflexi, can completely reduce TCE to ethene (Löffler et al., 2013; Yang et al., 2017). However, the effective use of *Dehalococcoides* to bioremediate the sites contaminated with chlorinated ethenes is limited to several factors, such as pH and the presence of co-existing electron acceptors.

The pH value significantly affects the TCE reductive dechlorination process. The pH range of *Dehalococcoides* is between 6 and 8, with the highest activity occurring between 6.9 and 7.5 (Löffler et al., 2013; Asai et al., 2022).

When the pH of the medium was 6.0, the dechlorination activity of *Dehalococcoides* decreased, leading to the accumulation of more toxic intermediate metabolites (DCE and VC). At a pH of 5.5, the dechlorination activity was lost (Yang et al., 2017; Ortiz-Medina et al., 2023). Reductive dechlorination and fermentation of organic substrates are acid-producing processes that lead to a decline in aquifer pH values (Brovelli et al., 2012; Robinson et al., 2009). Therefore, buffering techniques, including alkali addition and the use of non-acidified electron donors, are implemented to adjust and control aquifer pH to ensure the successful *in situ* bioremediation of contaminated sites (Borden et al., 2016; Lee et al., 2024). Sodium bicarbonate, or sodium carbonate, is often used as a buffer in the medium, but high bicarbonate concentrations can promote the growth of autotrophic methanogens and acetogens and consume large numbers of protons, resulting in an increase in the pH of the system. For example, when 10mM of HCO_3_^–^ was added to a medium with an initial pH of 7.5, the final pH rose to 8.7 (Borden et al., 2016; Delgado et al., 2012; Steffan and Environmental Security Technology Certification Program, 2010). In addition, insoluble silicate minerals and colloidal Mg(OH)_2_ were tested as long-term sources of alkalinity to control pH, while silicate minerals showed an inhibitory effect on the reductive dechlorination of DCE and VC. Conversely, colloidal Mg(OH)_2_ was successfully employed to control groundwater pH for over 5 years (Hiortdahl and Borden, 2014; Lacroix et al., 2014; Ortiz-Medina et al., 2023). Furthermore, sodium formate was used as an electron donor to minimize acidification, and a blend of emulsified substrate and modified rice husk ash was used as a green and long-lasting substrate (GLS) for slow carbon release and pH control (Lee et al., 2024; Philips et al., 2013; Tomita et al., 2022). However, the use of formate and GLS as pH buffers may be incapable of neutralizing H^+^ produced by high dechlorination rates because of the slow-release process. While exogenous substrates are added, natural buffering processes such as nitrate reduction also affect aqueous pH. However, their influence on reductive dechlorination remains unreported.

Nitrate (NO_3_^−^), a frequent co-contaminant with TCE, is mainly used in rocket propellants, explosives, and agricultural fertilizers (Carrey et al., 2021). In China, the highest nitrate concentration was up to 265 mg/L, and the detection rate of TCE was approximately 12% in some urban areas because of the unreasonable treatment of petroleum and textile pollution and the abuse of pesticides and fertilizers (Liu et al., 2017). On a global scale, the detected concentration of TCE ranged from 110 to 310 μg/L, and the maximum contaminant level of nitrate in the majority of wells was between 20 mg/L and 45 mg/L in the Fresno-Clovis metropolitan area (Kloos, 1997). In some industrial areas in Canada, such as Barrie-DC, Halifax Regional Municipality, and Greenwood, the detected concentrations of TCE and nitrate can reach up to 41 mg/L and 35 mg/L, respectively (Roy and Bickerton, 2012). NO_3_^−^ can be reduced to N_2_ through denitrification by denitrifying bacteria with organic matters (such as methanol, acetic acid, ethanol, etc.) or inorganic matters (such as hydrogen (H_2_), elemental sulfur, zero-valent iron, etc.) as electron donors (Rittmann and McCarty, 2001; Zhao et al., 2011). In the entire denitrification process, 5 moles of electrons are required for 1 mole of NO_3_^−^ reduction. There are many types of denitrifying bacteria, including more than 50 genera and 130 species, which are distributed among *α*, *β*, *γ*-*Proteobacteria*, *Deferribacteres*, *Firmicutes,* and *Actinobacteria* (Stein and Klotz, 2016; Zhao et al., 2013; Zhong et al., 2017).

The denitrification process is a self-producing alkali process, and 1 g of nitrate or nitrite consumption produces 3.57 g of basicity (calculated as CaCO_3_). In the high-load denitrification process, the pH value of the reaction solution can easily exceed 9.0 and inhibit the activities of denitrifying functional bacteria (Li et al., 2016; Shi et al., 2023). Many studies have found that nitrate inhibits TCE dichlorination, and the higher the nitrate concentration, the stronger the inhibitory effect (Cao et al., 2017; Xu et al., 2018; Zhang et al., 2022). Yin et al. (2018) verified that the denitrification byproduct N_2_O reacted with the super-reduced Co(I)-corrinoids of reductive dehalogenases to strongly inhibit the dechlorination activity. However, the pH adjustment by the interaction between nitrate self-alkalization and TCE self-acidification, along with their effects on nitrate reduction and reductive dichlorination, has not been studied.

This study established a series of experiments to evaluate the interaction between TCE and nitrate with or without external buffering substrates in mixed anaerobic dechlorinating consortia. TCE reductive dechlorination efficiencies, nitrate reduction, pH variation, and bacterial community structures were thoroughly investigated. The objectives of the present study were: (i) to explore the simultaneous reduction of nitrate and TCE under different pH conditions; (ii) to determine the effect of nitrate self-alkalization on the reductive dechlorination efficiency; and (iii) to reveal the changes in microbial community composition.

## 2 Materials and methods

To evaluate the interaction between TCE and nitrate, we conducted a set of experiments in serum bottles. The serum bottles contained microbial consortia that were capable of reducing TCE and/or nitrate. The bottles were amended with lactate as an electron donor and TCE and nitrate at different initial concentrations. To achieve the three objectives, we measured the concentrations of TCE, nitrate, and pH values during the experiments. We also studied the microbial community structure to further understand the interaction between TCE and nitrate from a microbial perspective. The experiment details are described below.

### 2.1 Description of the TCE-reducing consortium

The TCE-reducing consortium YH was subcultured from the YCQ1 culture (Kranzioch et al., 2013; Wen et al., 2017), grown using lactate as the sole electron donor, and maintained steadily for 6 years in the laboratory. *Dehalococcoides* spp. dominated the culture and could completely reduce 0.3 mM of TCE to non-toxic ethene in 20 days. The culture was maintained under anaerobic conditions and incubated in the dark at 30°C.

### 2.2 Interaction between nitrate and TCE in YH consortia

The reduced anaerobic medium for TCE reduction was prepared following the protocol outlined by Wen et al. (2020). The mineral salt medium contained the following reagents (per liter): 3.17 g of KH_2_PO_4_, 14.33 g of Na_2_HPO_4_•12H_2_O, 0.45 g of (NH_4_)_2_HPO_4_, 0.04 g of MgHPO_4_•3H_2_O, 1 mL of trace element solution A, and 1 mL of trace element solution B, as described by Kranzioch et al. (2013). In addition, 0.2 mM of L-cysteine and 0.2 mM Na_2_S•9H_2_O were added as the reducing agents. Resazurin was used as a redox indicator (Amos et al., 2008). Two types of media were prepared according to the requirements for buffer addition. Type I medium did not receive any buffering agents, and the initial pH was adjusted to 7.0 ± 0.2 by 1 M NaOH and 10% (vol/vol) HCl, while type II medium contained NaHCO_3_ and *tris*-ethanesulfonic acid (TES) to maintain the initial pH value below 6.5, which is referred to as suboptimal pH conditions. We transferred 75 mL of medium to 120 mL glass serum bottles under a stream of nitrogen (N_2_) and sealed the serum bottles with butyl rubber stoppers and aluminum crimps. When the color of the medium changed from pink to clear, we autoclaved the serum bottles in an inverted position.

To test the effect of nitrate on TCE dechlorination based on pH control by self-alkalization, the interaction experiment of nitrate and TCE bioreduction was conducted in the type-I mixed consortia. We added 25.5 mg/L (0.3 mM) of sodium nitrate, 39.4 mg/L (0.3 mM) of TCE, 25.5 mg/L (0.3 mM) of sodium nitrate +39.4 mg/L (0.3 mM) of TCE, and 255 mg/L (3 mM) of sodium nitrate +39.4 mg/L (0.3 mM) of TCE to four bottles. The bottle with a 1:1 molar concentration ratio of TCE and nitrate was named T1N1, and the bottle with a 1:10 molar concentration ratio was named T1N10. The bottle with only TCE or nitrate was named T1 or N1, respectively. Each bottle was incubated with 5 mL of bacterial solution from the YH consortium. Samples for TCE and nitrate analysis were collected periodically: every 8 h during TCE reduction and every 2 days thereafter. All experiments were conducted with duplicate bottles. The results are presented as the average values of the duplicates.

To further investigate the ability of nitrate self-alkalization to regulate pH and its effect on reductive dechlorination under suboptimal pH conditions, similar treatments were applied to the type-II mixed consortia. The experimental setups were as follows: the bottle containing equal molar concentrations of TCE and nitrate was named Buffer-T1N1, while the bottle with 0.3 mM of TCE and 3 mM of nitrate was named Buffer-T1N10. Bottles containing only TCE or nitrate were named Buffer-T1 or Buffer-N1, respectively. All experiments were conducted in duplicate, and the results are presented as the average values of the two replicates.

### 2.3 Chemical analyses

TCE, *cis*-DCE, VC, ethene, and methane were measured by injecting 100 μL of headspace samples (with a gas-tight syringe) into a gas chromatograph (Agilent Technologies GC system, model 6,890 N, Agilent Technologies Inc., United States) equipped with a flame-ionization detector (FID) and a packed column (RT-SQ-Bond, 30 m long, 0.53 mm i.d., 20 μm thickness, Restek, United States). N_2_ was used as the carrier gas, fed at a constant flow rate of 86.9 mL/min. The temperature conditions for the injector and detector were 200 and 240°C, respectively. The program consisted of the following steps: initially holding the temperature at 60°C for 2 min, then heating gradually to 100°C (10°C/min), holding for 2 min, and finally heating gradually to 210°C, and holding at 210°C for 6 min. Analytical-grade chloroethene, ethene, and methane were added into 80 mL of water in 120 mL bottles to provide standards for calibration curves that were linear (*R*^2^ ≥ 0.996). Ethene and methane concentrations in the liquid were computed using their Henry’s constants (*K*_H_): ${\left[\mathrm{Compound}\right]}_{liq}={\left[\mathrm{Compound}\right]}_{\mathrm{gas}}/K_{H}$ .

The calculated dimensionless Henry’s constants (mM_gas_/ mM_liq_, *T* = 25°C) used in this study were 8.35 for ethene and 28.99 for methane.

Volatile fatty acids (VFAs) containing lactate, acetate, and propionate were analyzed by injecting 10 μL of liquid samples into high-performance liquid chromatography (HPLC, LC-16, Shimadzu Corporation) with an ultraviolet detector, an organic acid column (ECOSIL Sugar H^+^, 10 μm, 7.8 × 300 mm), a mobile phase of 0.5 mM H_2_SO_4_, and a 0.6 mL/min flow rate. The detection wavelength was 210 nm. 1 mL of liquid samples was filtered through a 0.22-μm polyvinylidene fluoride membrane syringe filter into 2 mL glass vials for subsequent analysis. Calibration curves were generated for all VFA analyses during each HPLC run. The detection limits for VFAs on the HPLC were 0.1 mg/L. NO_3_^−^ was measured according to a standard method. A pH meter was used to monitor the pH value (DZS-706F, Leighz, Shanghai, China).

### 2.4 Electron distribution analysis

The electron distribution for each reaction was calculated as described previously (Delgado et al., 2012; Wen et al., 2015). The numbers of e- equivalents (eq) required for the dechlorination of TCE per mole are 2, 4, and 6 to DCE, VC, and ethene, respectively, with the reduction of each mole of nitrate to N_2_ needing 5 e- eq, and each mole of lactate providing 12 e^−^ eq. The electron distributions were calculated as follows:


$$\%\mathrm{Compound}=\frac{\left(\mathrm{Compound}\right)\times \frac{\mathrm{electrons}}{\mathrm{mole}}}{\left(H_{2}\right)\times \frac{2\mathrm{electrons}}{\mathrm{mole}\;H}}\times 100$$

The related reactions and equations are listed in Supplementary Table S1.

### 2.5 Molecular analysis

At the end of the operation, 20 mL of liquid samples without buffering agents were centrifuged for 30 min at 8000 rpm (5,900 g) at 4°C (Eppendorf 5415R, Germany) with Falcon tubes. The pellet samples were sent to BiOWEFIND Technology (Wuhan, China) to perform Illumina MiSeq sequencing with standard protocols, including amplicon generation, which used primers 341F (5′-CCTAYGGGRBGCASCAG-3′) and 806R (5′-GGACTACNNGGGTATCTAAT-3′) to target the conserved V3 to V4 regions of the bacterial 16S rRNA gene (Caporaso et al., 2010a). The data were processed using the QIIME (version 1.7.0) pipeline (Caporaso et al., 2010b).

## 3 Results

### 3.1 Interaction between TCE and nitrate bioreduction under different pH conditions

**Figure 1:**
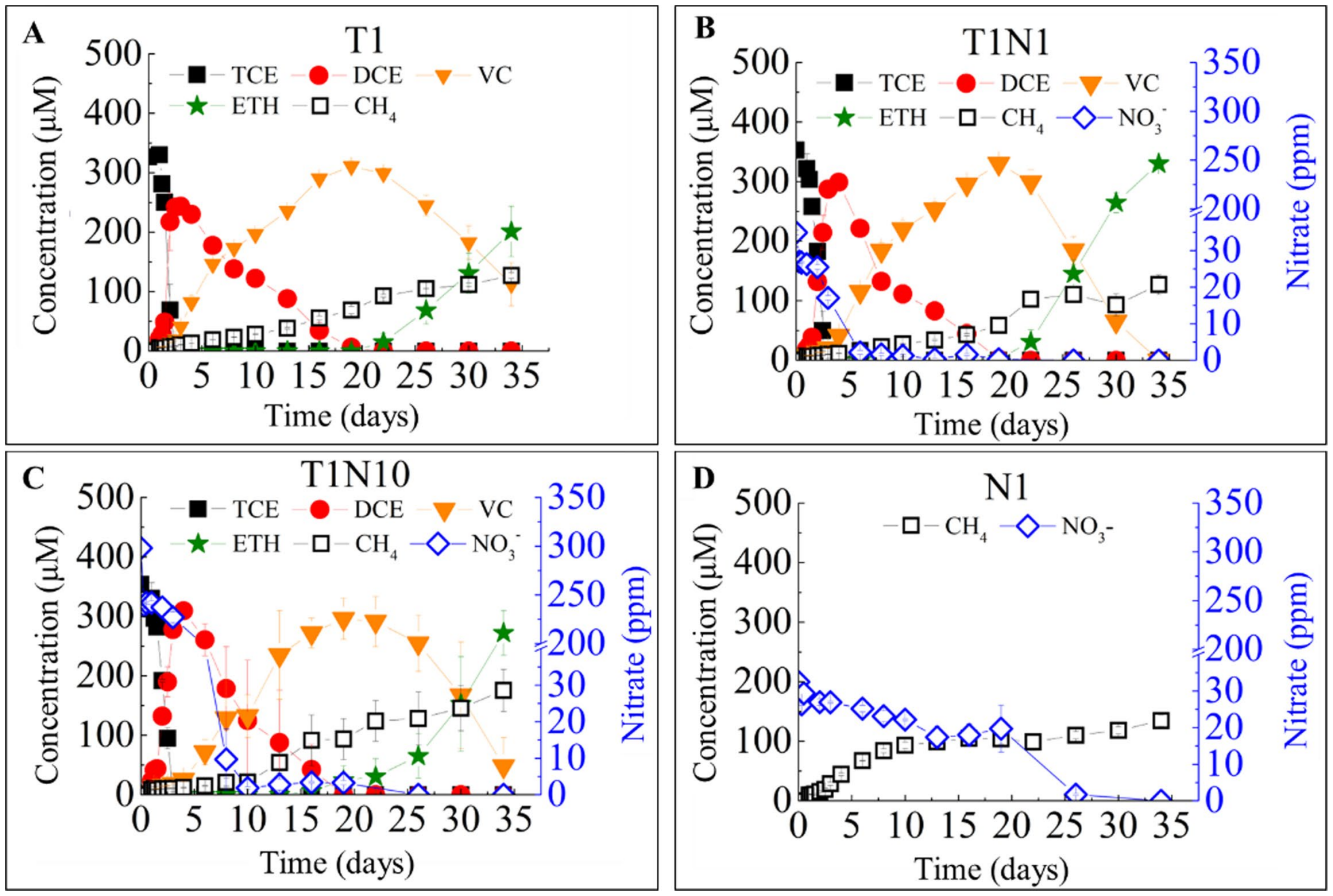
The TCE and nitrate bio-reductions on the TCE- dechlorinating cultures without buffering agents in a batch test. T1 **(A)** means the culture added with TCE as sole electron acceptor, the concentration ratio of TCE and nitrate was 1:1 in the culture T1N1 **(B)**, whereas 1:10 in the culture T1N10 **(C)**. and N1 **(D)** means the culture added with nitrate as sole acceptor.

Figure 1 illustrates the reduction of TCE and/or nitrate by the dechlorinating consortia under neutral pH conditions without buffering agents. The nitrate reduction affected the TCE reductive dechlorination stepwise. The conversion rate of TCE to *cis*-DCE decreased from 130.6 ± 0.3 μmol Cl^−^/(L·d) in the positive control batch (T1) to 121.4 ± 0.4 μmol Cl^−^/(L·d) in the presence of 0.3 mM nitrate (T1N1) and further to 103.6 ± 0.2 μmol Cl^−^/(L·d) with 3 mM nitrate (T1N10).

In TCE-amended batches with varying initial nitrate concentrations, *cis*-DCE was fully reduced to VC in 19 days. However, the presence of nitrate significantly enhanced VC reduction to ethene to varying degrees (*p* = 0.016, Table 1). VC was completely converted to ethene in the presence of 0.3 mM (25.5 ppm) nitrate (T1N1), while 78.4% of VC was reduced in the presence of 3 mM (225 ppm) nitrate (T1N10), compared to 63.9% in the T1 batch without nitrate throughout the experiment.

**Table 1:** Two-way analysis of variance of different concentration of nitrate and TCE on the chlorinated ethenes and nitrate reduction.

Factors	*k_TCE-DCE_*		*k_DCE-VC_*		*k_VC-ETH_*		Nitrate removal rate	
	*F*	*p*	*F*	*p*	*F*	*p*	*F*	*p*
Nitrate	1.154	0.387	4.143	0.087	10.513	0.016^*^	139.684	0.000^**^
Buffer	34.989	0.002^*^	1.852	0.232	36.313	0.002^*^	0.095	0.771
TCE	–	–	–	–	–	–	4.720	0.083
Nitrate × Buffer	1.134	0.336	0.021	0.890	0.609	0.470	–	–

* and ** mean the results have a significant influence when *p* ≤ 0.05 and *p* ≤ 0.01, respectively.

Nitrate was completely reduced in all the batches amended with nitrate. The reduction of TCE stimulated nitrate conversion. In the presence of 0.3 mM nitrate, the reduction time decreased from 25 days in the culture with nitrate as the sole electron acceptor (N1) to only 6 days in the T1N1 batch. When the concentration of nitrate increased 10-fold, the nitrate reduction rate significantly increased from 5.5 mg/(L·d) in T1N1 to 31.9 mg/(L·d) in T1N10.

Figure 1 also shows the formation pattern of methane. 0.3 mM TCE had an inhibitory effect on methanogenesis. In the N1 treatment with only 0.3 mM nitrate added, the methane concentration increased from 0 to 93.2 μmol/L at a rate of 9.3 μmol/(L·d) within 10 days, while the formation rate slowed down [1.7 μmol/(L·d)], and the final concentration was 134.6 μmol/L. However, methane production was severely inhibited in consortia containing TCE. The methane formation pattern was similar in the treatment group supplemented with only TCE and TCE and 0.3 mM nitrate. On the 10th day, the methane content was ~20 μmol/L and gradually increased after 10 days, with the final concentration of 127.1 μmol/L. In the treatment with 3 mM nitrate added, the activity of methanogens became stronger after 10 days, and the methane concentration increased from 21.3 μmol/L to 175.3 μmol/L at a rate of 6.4 μmol/(L·d).

**Figure 2:**
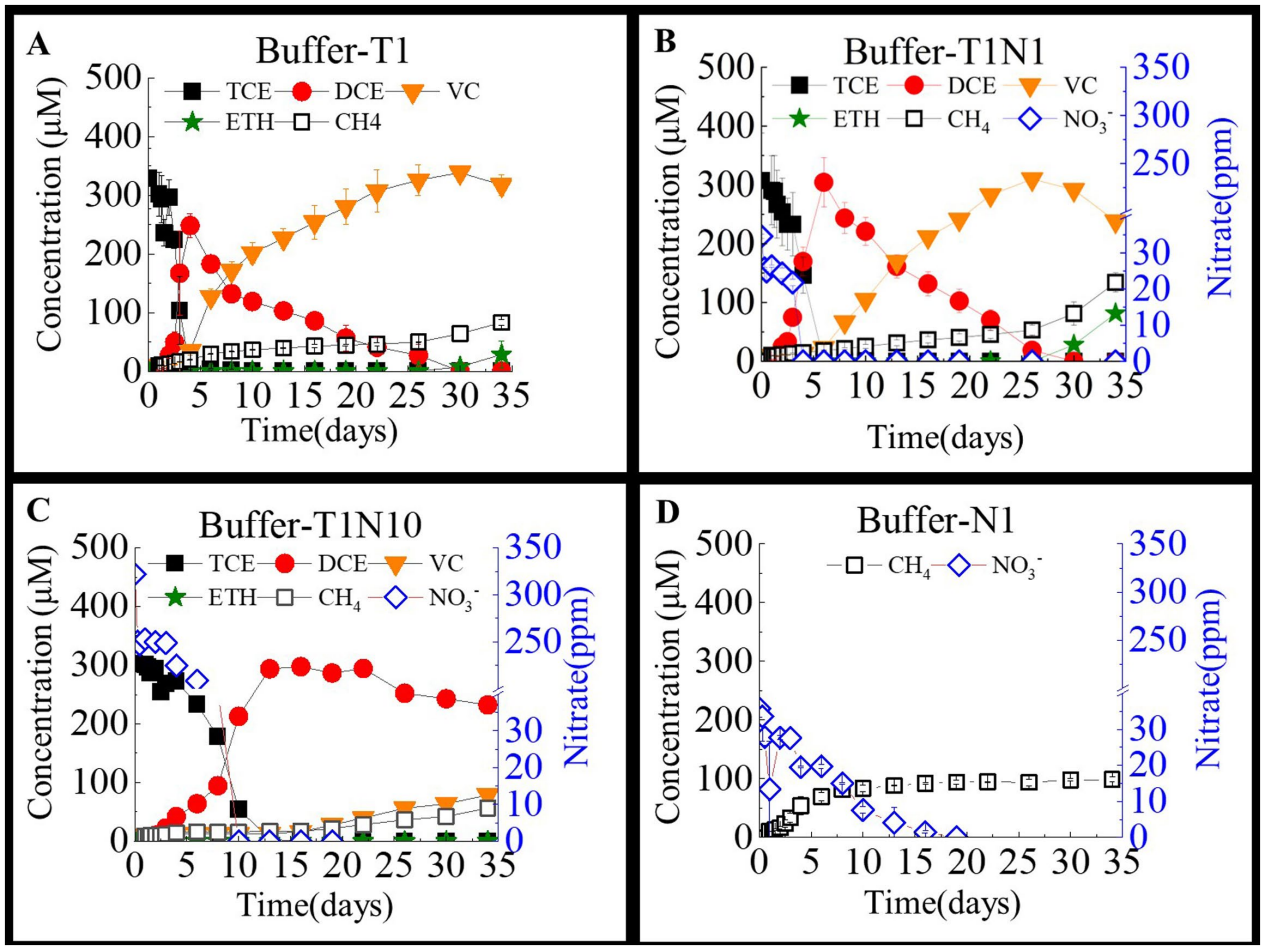
The TCE and nitrate bio-reductions on the TCE- dechlorinating cultures amended with bicarbonate and tris-ethanesulfonic acid as buffering agents in a batch test. Buffer-T1 **(A)** means the culture added with TCE as sole electron acceptor, the concentration ratio of TCE and nitrate was 1:1 in the culture Buffer -T1N1 **(B)**, whereas 1:10 in the culture Buffer-T1N10 **(C)**. and Buffer-N1 **(D)** means the culture added with nitrate as sole acceptor.

Figure 2 shows the interaction of nitrate and TCE reduction in acidic consortia with NaHCO_3_ and *tris*-ethanesulfonic acid added as buffering agents to maintain the pH below 6.5. TCE reduction was significantly inhibited by nitrate in acidic consortia compared with neutral treatments, as shown in Figure 1 and Table 1. In the culture containing only TCE as an electron acceptor, TCE was fully transformed into DCE within 4 days. Then, DCE was completely reduced to VC at a dechlorination rate of 9.5 μmol/(L·d) on day 30. At the end of the experiment, only approximately 8.98% of the VC was converted to ethane. In contrast, when the nitrate concentration increased from 0 to 0.3 mM and 3 mM, the dechlorination of TCE to cDCE required more than 6 days and 13 days, respectively. When the concentration of nitrate was 0.3 mM, the reduction of DCE and VC was similar to that of the TCE-only group, and DCE was completely reduced to VC at a dechlorination rate of 12.7 μmol/(L·d), with 81.5 μmol/L of ethene detected at the end of the experiment. However, when the concentration of nitrate was increased to 3 mM, the reduction of DCE was severely inhibited. At the end of the experiment, only 21.9% of DCE was reduced to VC, and no ethene was detectable. In comparison, the acidic environment was more favorable for nitrate reduction than the neutral environment. In the culture with only added nitrate, 95.8% of the nitrate was reduced within 16 days. In the culture with coexisting TCE, nitrate was completely reduced within 5 days at a rate of 8.7 mg/(L·d). When the nitrate concentration was increased to 3 mM, the nitrate reduction rate increased to 32.2 mg/(L·d).

### 3.2 Variation of pH during TCE and nitrate bio-reduction

**Figure 3:**
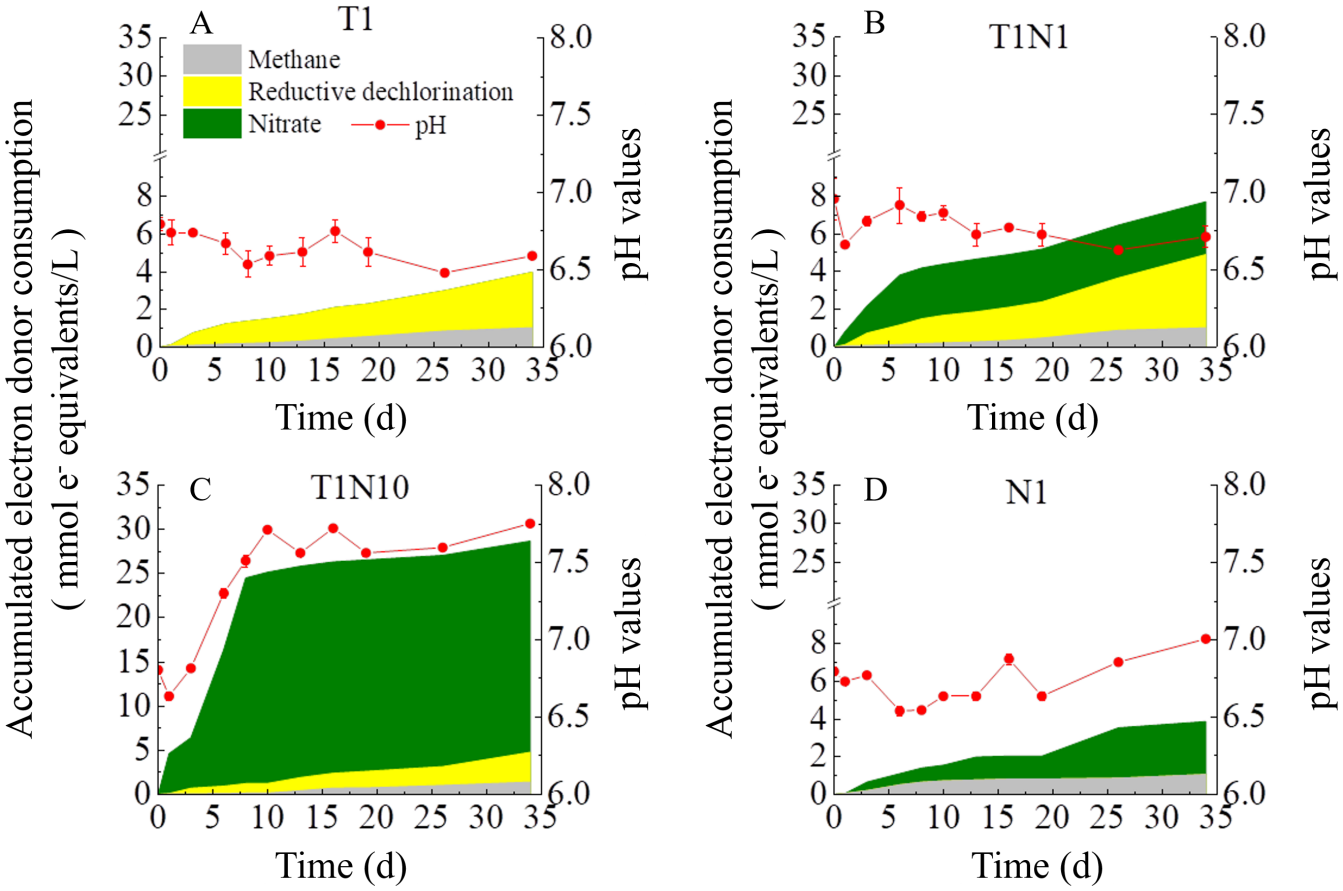
The accumulative electron donor consumption and pH variation in the mixed dechlorinating culture without buffering agents. The culture added with TCE as sole electron acceptor was named T1 **(A)**, the concentration ratio of TCE and nitrate is 1:1 in the culture T1N1 **(B)**, whereas 1:10 in the culture T1N10 **(C)**. and N1 **(D)** means the culture added with nitrate as sole acceptor.

Figure 3 shows the variation of pH in different consortia using HCl/NaOH to make the medium neutral (pH 6.8–7.2) before inoculation. The shift in pH values distinguished in the presence or absence of nitrate and ranged from 6.5 to 7.0, except for the T1N10 culture. The pH decreased from the initial 6.80 ± 0.04 to the final 6.59 ± 0.1 in culture T1 fed with TCE as the only electron acceptor. With simultaneous addition of TCE and nitrate, pH first dropped from 7.00±0.14 to 6.66±0.02 on day 1, then increased to 6.92±0.12 on day 5, and finally gradually declined to 6.71±0.07 at the end of the experiment in the culture T1N1. When the concentration of nitrate was increased by 10-fold, the pH values gradually increased from 6.64 ± 0.03 to 7.71 ± 0.01 from day 1 to day 10 and were maintained at 7.65 ± 0.09 steadily until the end of the experiment in the culture T1N10. In the culture N1 amended with nitrate as the sole electron acceptor, the pH decreased from an initial 6.80 ± 0.02 to 6.64 ± 0.02 on day 20, then increased to 7.01 ± 0.01 at the end of the experiment.

In the presence of buffering agents, the pH values did not change significantly due to the reduction of 0.3 mM TCE and/or 0.3 mM nitrate. However, in the culture amended with 3 mM nitrate, the pH value significantly increased from 6.27 ± 0.03 on day 8 to 6.69 ± 0.10 at the end of the experiment (data was shown in Supplementary Figure S1).

### 3.3 Electron donor utilization

**Figure 4:**
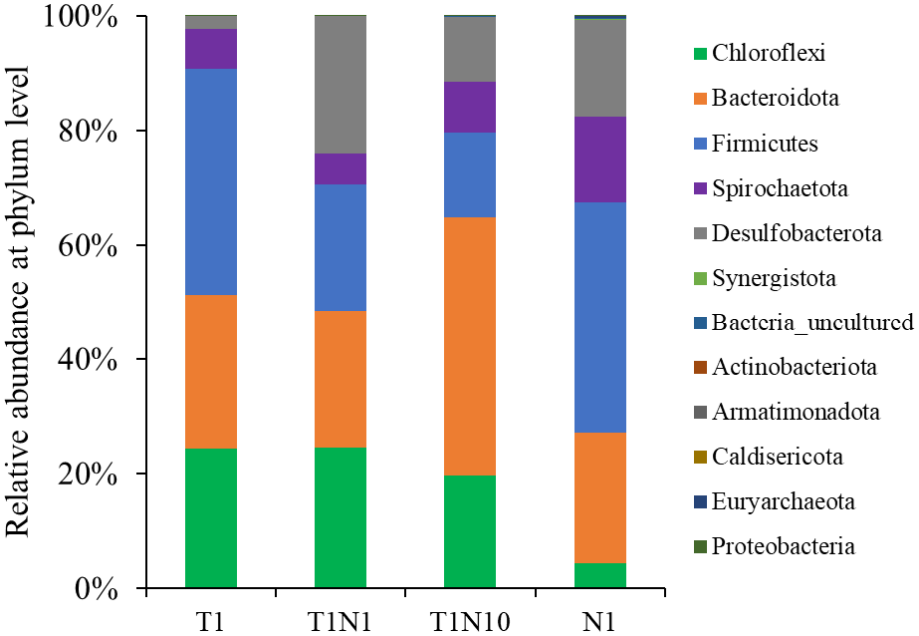
The microbial community structure of tested consortia at the end of experiments at phylum level.

All cultures were provided with sufficient lactate as an electron donor and carbon source throughout the whole experiment. Lactate was instantly fermented to acetate and propionate (Supplementary Figure S2). Figure 3 shows the consumption of accumulated electron equivalents by the three main reactions (reductive dechlorination (RD), nitrate reduction, and methanogenesis). The electron utilization was calculated using the method described in Ziv‐El et al. (2012) (Supplementary Table S1). The total amounts of electron donor consumption increased with the concentration of nitrate. The ratios of electron equivalents consumed by nitrate reduction and RD (nitrate/RD) were 0.72 and 7.08 under 25.5 and 255 mg/L (0.3, 3 mM) of nitrate, respectively. Figure 4 also shows that methane formation consumed a certain amount of electrons. The ratios of electron equivalents consumed by methanogenesis and RD (methane/RD) were 0.26 and 0.41 under 25.5 and 255 mg/L (0.3, 3 mM) of nitrate, respectively.

### 3.4 The shifts in microbial community structure

Figure 4 shows the microbial community structure of the cultures at the phylum level. The major microbial members were similar in dechlorinating consortia fed with different concentrations of TCE and/or nitrate. Notably, Firmicutes (14.81–40.38%), Bacteroidota (22.86–45.04%), Chloroflexi (4.27–24.46%), Spirochaetota (5.32–14.82%), and Desulfobacterota (2.23–23.97%) were the most dominant. In TCE-containing cultures, the Chloroflexi abundance increased to 24.46% after adding the 0.3 mM nitrate but decreased to 19.72% with 3 mM nitrate. However, when the culture was amended with nitrate alone as an electron acceptor, the relative abundance of Chloroflexi decreased to 4.3%. Along with increasing nitrate concentrations, the proportion of Firmicutes dropped, reaching its lowest abundance of 14.8% at 3 mM nitrate. In contrast, the proportions of Desulfobacterota significantly increased from 2.2 to 24.0 and 11.2%, respectively. Bacteroidota was considerably enriched, and the proportion was 45.0% in the presence of 3 mM nitrate.

**Figure 5:**
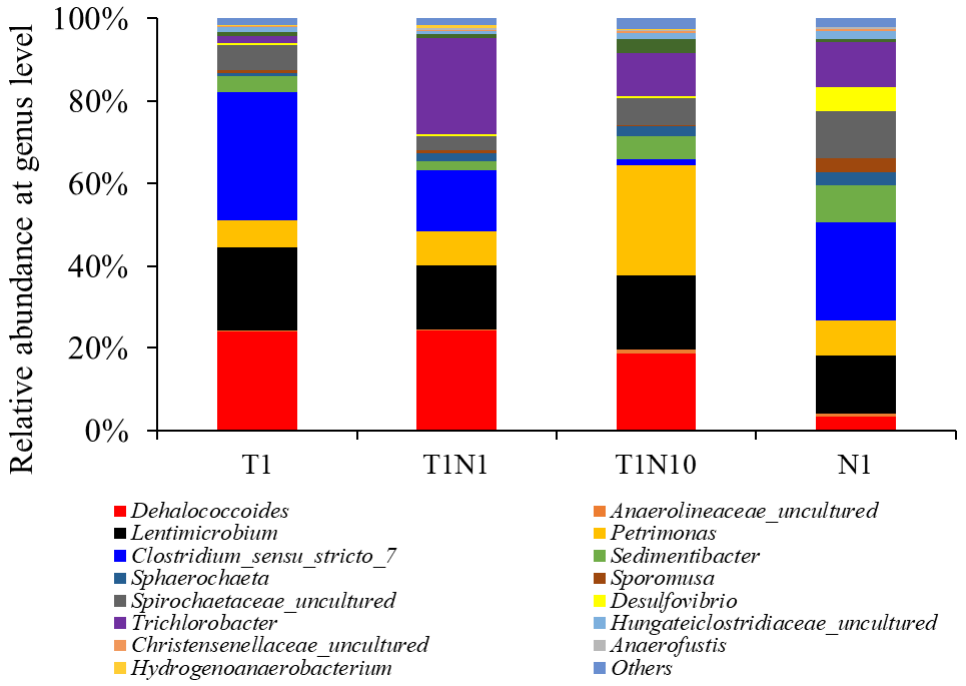
The microbial community structure of tested consortia at the end of experiments at genus level.

The trend of microbial community structure in the cultures at the genus level was consistent with the change in relative abundance at the phylum level (Figure 5). In the TCE-containing consortia, the relative abundance of *Dehalococcoides* belonging to the phylum of Chloroflexi increased slightly from 23.9 to 24.26% when exposed to 0.3 mM nitrate but decreased to 18.6% in the presence of 3 mM nitrate. *Clostridium* was the most dominant in the positive culture, but its abundance dramatically declined from 31.11 to 1.51% when nitrate concentration increased from 0 to 3 mM. The abundance of *Petrimonas* rose with the addition of increasing doses of nitrate, accounting for 6.53, 8.34, and 16.73% when exposed to 0, 0.3, and 3 mM nitrate, respectively. Meanwhile, the presence of nitrate activated the growth of *Trichlorobacter* to varying degrees, increasing from 1.69 to 23.27 and 10.57% in the presence of 0.3 and 3 mM nitrate, respectively. *Desulfovibrio* and *Sporomusa* were present at less than 1% in the TCE-containing cultures, but were prevalent in the culture supplemented with nitrate as the sole electron acceptor, accounting for 5.85 and 3.23%, respectively. Among all the cultures, *Lentimicrobium*, *Spirochaetaceae*, and *Sedimentibacter* were dominant, with abundances ranging from 2.08 to 20.33%.

## 4 Discussion

We conducted a series of experiments to investigate the interaction between TCE and nitrate bioreduction under varying pH conditions. This information is essential for the bioremediation of chlorinated ethenes and nitrate, which are generally present as co-contaminants in contaminated sites. We found that nitrate reduction affected reductive dechlorination relatively differently under pH conditions without external buffering agents.

The transformation of TCE to *cis*-DCE was inhibited by nitrate reduction, which is similar to previous studies. For example, Fallahpour et al. (2018) demonstrated that TCE remediation efficiency dropped by approximately 10% in the presence of 40 mg/L (0.47 mM) nitrate compared to 2 mg/L (0.02 mM). Similarly, Zhang et al. (2022) reported that TCE removal efficiency decreased from 95.28 ± 0.4% to 85.98 ± 0.2% when the nitrate concentration increased from 25 mg/L (0.29 mM) to 100 mg/L (1.18 mM).

This inhibition of TCE degradation to *cis*-DCE may be attributed to the toxic effects of nitrate or denitrification byproducts, nitrite, and N_2_O, on dechlorinating populations. Both nitrite and N_2_O are known inhibitors of reductive dehalogenases and have been confirmed to strongly inhibit dechlorination activity (Neumann et al., 1995; Suyama et al., 2001; Yin et al., 2018).

Cao et al. (2017) also indicated that nitrate strongly inhibits the reductive dechlorination of 2,4,6-trichlorophenol, while Boopathy and Peters (2001) observed that under nitrate-reducing conditions, reductive dechlorination ceased at *cis*-DCE and *trans*-DCE. However, the conversion of *cis*-DCE to VC was not significantly affected in this study. The inhibitory effect of nitrate on dechlorination diminished once it was fully reduced (Cao et al., 2017; Zhang et al., 2022).

Notably, contrary to previous findings, we found that the conversion of VC to ethene was enhanced by nitrate reduction. This enhancement may be linked to pH changes associated with nitrate reduction. In mixed dechlorinating consortia, processes such as reductive dechlorination and lactate fermentation produce H^+^, which acidifies the solution. In contrast, nitrate reduction is a self-alkalizing process that alkalizes the solution.

In our experiments, TCE was added as the sole electron acceptor without buffering substrates. The pH in cultures without nitrate gradually decreased due to fermentation and reductive dechlorination, leading to a decline in the TCE reduction rate. This decrease is critical as *Dehalococcoides*, the genus responsible for converting VC to ethene, is highly sensitive to pH, with an optimal growth range of 6.9 to 7.5 (Lee et al., 2024; Yan et al., 2021; Yang et al., 2017). In cultures with added nitrate, the pH increased as nitrate was reduced. This effect was particularly pronounced at a nitrate concentration of 3 mM, where the pH increased to 7.75 by the end of the experiment. These findings indicate that nitrate can function as a buffer to stabilize pH and enhance dechlorination rates. However, a nitrate concentration of 0.3 mM proved to be more effective than 3 mM for promoting the dechlorination process. This highlights the potential for leveraging nitrate self-alkalization in optimizing bioremediation strategies.

*In situ,* contaminated sites often exhibit acidic conditions due to natural factors such as high concentrations of dissolved carbon dioxide, desorption of H^+^ from clay minerals, or human-influenced causes such as acid rain (Ortiz-Medina et al., 2023). In regions with higher rainfall, pH values below 6 are commonly observed (Zhou et al., 2015).

To further explore the potential of nitrate self-alkalization to adjust pH under suboptimal conditions, simultaneous reduction experiments were conducted using NaHCO_3_ and *tris*-ethanesulfonic acid as buffers to maintain the initial pH below 6.5. We found that the reduction of 0.3 mM nitrate effectively mitigated the pH decrease and enhanced the reduction rate of VC to ethene. However, at a nitrate concentration of 3 mM, despite the pH reaching the optimal growth range, the dechlorination process was significantly inhibited, leaving 74.73% DCE and 25.27% VC as the final products.

These findings confirmed that nitrate reduction can increase pH even in the presence of buffering substrates. Under suboptimal pH conditions, a moderate nitrate concentration (e.g., 0.3 mM) slightly promoted the reductive dechlorination process. However, higher nitrate concentrations (e.g., 3 mM) severely inhibited dechlorination, emphasizing the importance of optimizing nitrate dosage for effective bioremediation.

As expected in the dechlorinating consortia, we observed nitrate reduction and methanogenesis. Methanogenesis was strongly inhibited in this study, which indicated that 0.3 mM TCE is toxic to methanogens. Various studies have shown that chlorinated ethenes are toxic to microorganisms. TCE (10 mg/kg) inhibited the process of nitrate transformation into nitrite by affecting the activity of nitrate reductase in Mollisol (Li et al., 2020). However, we found the tested TCE concentration had no inhibitory effect on the nitrate reduction, and the nitrate removal rate was faster in the presence of TCE than in the culture with nitrate as the sole electron acceptor. This may be due to the inoculated culture used in this study, which had never been exposed to nitrate before and needed an acclimation time to activate the process of denitrification.

Further electron distribution study showed that the electrons flowing to TCE reduction declined when the nitrate concentration increased. On the one hand, denitrifying bacteria are prevalent in the environment and have diverse metabolic pathways (Sun et al., 2024; Zhang et al., 2022). On the other hand, nitrate is a more thermodynamically favorable electron acceptor than TCE or its daughter products (Table 2), so the reductive dechlorination process can be easily outcompeted.

**Table 2:** The full reactions of electron acceptors and their Gibb’s standard free energy at pH = 7.0.

	Full-reactions	ΔG^0’^/kJ/e^−^ eq
Nitrate^a^	1/5 NO_3_^−^ + 1/5 H^+^ + 1/2 H_2_ = 1/10 N_2_ + 3/5 H_2_O	−118.59
CO_2_^a^	1/8 CO_2_ + 1/2 H_2_ = 1/8 CH_4_ + 1/4 H_2_O	−16.34
TCE^a^	1/2 C_2_HCl_3 (aq)_ + 1/2 H_2_ = 1/2 C_2_H_2_Cl_2 (aq)_ + 1/2 Cl^−^ + 1/2 H^+^	−34.66
DCE^a^	1/2 C_2_H_2_Cl_2 (aq)_ + 1/2 H_2_ = 1/2 C_2_H_3_Cl _(aq)_ + 1/2 Cl^−^ + 1/2 H^+^	−30.36
VC^a^	1/2 C_2_H_3_Cl _(aq)_ + 1/2 H_2_ = 1/2 C_2_H_4 (g)_ + 1/2 Cl^−^ + 1/2 H^+^	−31.59

a The full reactions and the calculation of Gibb’s standard free energy based on the free energy of formation for various species at 25°C cited from Environmental Biotechnology Principles and Applications (Rittmann and McCarty, 2001).

Based on the sequencing results, we found that *Dehalococcoides* belonging to Chloroflexi and putative acetogenic bacteria belonging to *Clostridium*_sensu_stricto_7, *Lentimicrobium,* and *Spirochaetaceae* dominated the consortia added with TCE as an electron acceptor. Many studies have shown the co-existence of *Dehalococcoides* and these fermenters, which stimulated *Dehalococcoides* growth by providing acetate and corrinoid cofactors (Wen et al., 2020; Yan et al., 2021). However, we found that the abundance of *Dehalococcoides* and *Clostridium* declined in cultures with higher concentrations of nitrate, revealing the inhibitory effect of nitrate on *Dehalococcoides* and *Clostridium*. While *Lentimicrobium* maintained a relatively high abundance in the presence of nitrate in this study. *Lentimicrobium* has been shown to grow in the pH range of 6.6–9.0 and has been shown to play a significant role in the reduction of high concentrations of nitrate (Li J. et al., 2022; Li Y. et al., 2022; Sun et al., 2016; Wang et al., 2020; Zhang et al., 2024). In comparison, we found that *Petrimonas* and *Trichlorobacter* dominated the cultures exposed to TCE and nitrate. Chen et al. (2023) showed that hydrogen production and DCE removal were positively correlated with *Petrimonas*. Grabowski et al. (2005) reported that *Petrimonas* can use nitrate as an electron acceptor.

Similarly, we found that the abundance of *Petrimonas* increased, especially when the nitrate concentration was up to 3 mM (255 mg/L). This implied that *Petrimonas* was potentially involved in the process of dechlorination and denitrification in the culture exposed to high concentrations of nitrate. *Trichlorobacter was* positively correlated with TCE dechlorination efficiency (Chen et al., 2023; De Wever et al., 2000). We found that the abundance of *Trichlorobacter* was negligible in the culture with TCE as the sole electron acceptor, but was dominant in the culture in the presence of nitrate. This indicates that *Trichlorobacter* is possibly responsible for nitrate reduction and outcompetes *Petrimonas* when nitrate concentrations are low. Much more research is needed to understand the mechanism of nitrate action at TCE-contaminated sites further.

## 5 Conclusion

We evaluated the interaction between nitrate and TCE through a series of experiments conducted in serum bottles. The results indicated that TCE concentrations up to 0.3 mM promoted nitrate reduction, while stepwise reductive dechlorination was differentially affected by nitrate under varying pH conditions. Although nitrate inhibited the reduction of TCE to DCE, an appropriate nitrate dose enhanced the conversion of VC to ethene. This outcome was mainly due to the thermodynamical preference of nitrate reduction compared to TCE reduction, while the enhancement of VC conversion was linked to pH stabilization by the self-alkalization process.

As the nitrate concentration increased from 0 to 3 mM, the relative abundance of fermentative *Clostridium* abundance sharply decreased from 31.11 to 1.51%, whereas putatively denitrifying genera *Petrimonas* and *Trichlorobacter* showed significant increases. Furthermore, when the nitrate concentration was increased to 0.3 mM, the relative abundance of *Dehalococcoides* slightly increased from 23.91 to 24.26%. However, at a nitrate concentration of 3 mM, *Dehalococcoides* abundance decreased to 18.65%, indicating a tolerance to high nitrate concentrations under specific conditions.

## Data Availability

The original contributions presented in the study are included in the article/supplementary material, further inquiries can be directed to the corresponding author.
