# Supplementary material for: Interaction between nitrate and trichloroethene bioreduction in mixed anaerobic cultures
Source: Front Microbiol. 2025 Jan 15;15:1504235. doi: 10.3389/fmicb.2024.1504235 (PMC11778175; doi:10.3389/fmicb.2024.1504235)
Supplement: Supplementary file 1 [file Data_Sheet_1.docx]

Cover sheet for SI

Authors:

Dong-Mei Yang^#^, Fen-Li Min^#^, Ying Li, Jia-Lu Ling, Hui-Xian Zhong, Yu-Chun Xia, Ying Feng, Li-Ya, Zhao, Zhao-Hua Li, Li-Lian Wen^*^

Manuscript title: Interaction of nitrate and trichloroethene bio-reduction in mixed anaerobic cultures

Number of pages: 6

Number of tables: 2

Number of figures: 2

[Supplementary information](http://www.rsc.org/suppdata/dt/b9/b924945c/b924945c.pdf) (SI) for manuscript

**Interaction of nitrate and trichloroethene bio-reduction in mixed anaerobic cultures**

Dong-Mei Yang^1#^, Fen-Li Min^2#^, Ying Li^1^, Jia-Lu Ling^1^, Hui-Xian Zhong^1^, Yu-Chun Xia^1^, Ying Feng^1^, Li-Ya, Zhao^1^, Zhao-Hua Li^1^, Li-Lian Wen^1*^

1. College of Resource and Environmental Science, Hubei University, Wuhan 430062, China;

2. Hubei Key Laboratory of Environmental and Health Effects of Persistent Toxic Substances, School of Environment and Health, Jianghan University, Wuhan 430056, China

# Share the first author

* Correspondence to Dr. Li-Lian Wen. Tel (Fax): 027-88661699, E-mail: wll@hubu.edu.cn

**Table S1.** Electron-donor distribution based on method of Ziv-El et al. (2012).

| Steady-state end product(mM) | Biochemical Process | $\frac{me^{-}eq}{mmol}$ | steady-state end product ( )  $\frac{me^{-}eq}{L}$ |
| --- | --- | --- | --- |
| [*cis*-DCE],[VC],[ethene] | 1) C_2_HCl_3_+3H_2_=C_2_H_4_+3Cl^-^+3H^+^ | 6 | [*cis*-DCE]×2+[VC]×4+[ethene]×6 |
|  | 2) C_2_HCl_3_+2H_2_=C_2_H_3_Cl+2Cl^-^+2H^+^ | 4 |  |
|  | 3) C_2_HCl_3_+H_2_=C_2_H_2_Cl_2_+Cl^-^+H^+^ | 2 |  |
| [Nitrate] | 1) NO_3_^-^ + H_2_ = NO_2_^-^ + H_2_O | 5 | [Nitrate]×5 |
|  | 2) NO_2_^-^ + 3/2H_2_  = 1/2N_2_ + H_2_O + OH^-^ |  |  |
| [Methane] | 1) HCO_3_^-^ +4H_2_+H^+^=CH_4_+3H_2_O | 8 | [Methane]×8 |
| [Lactate] | 1) CH_3_CH_2_OCOO^-^+4H_2_O = 2CO_2_+HCO_3_^-^+6H_2_ | 12 | [Lactate]×12 |
| [Acetate] | 1) CH_3_COO^-^ + 3H_2_O = CO_2_ + HCO_3_^-^ + 4H_2_ | 8 | [Acetate]×8 |
| [Propionate] | 1) CH_3_CH_2_COO^-^ + 5H_2_O = HCO_3_^-^ + 2CO_2_ + 7H_2_ | 14 | [Propionate]×14 |

**Table S2.** Transformation rates of three main reactions (reductive dechlorination (RD), nitrate reduction, and methanogenesis) in the mixed anaerobic consortia.

| Group | TCE→*cis*-DCE  (μmol Cl^-^/(L·d)) | *cis*-DCE→VC  (μmol/(L·d)) | VC→ETH  (μmol/(L·d)) | Methane generation  (μmol/(L·d)) | NO_3_^-^ reduction  (mg/(L·d)) |
| --- | --- | --- | --- | --- | --- |
| T1 | 130.6±0.3 | 14.9±0.1 | 13.2±0.2 | 3.7±0.0 | — |
| T1N1 | 121.4±0.4 | 19.8±0.2 | 22.0±0.1 | 3.7±0.1 | 5.5±0.0 |
| T1N10 | 103.6±0.2 | 20.6±0.2 | 16.5±0.1 | 6.4±0.0 | 31.9±0.0 |
| N1 | — | — | — | 9.3±0.0 | 1.2±0.0 |
| Buffer-T1 | 81.5±0.2 | 9.5±0.0 | 5.3±0.1 | 2.4±0.1 | — |
| Buffer-T1N1 | 51.2±0.1 | 12.7±0.0 | 8.9±0.1 | 3.9±0.0 | 8.7±0.0 |
| Buffer-T1N10 | 23.0±0.3 | 8.1±0.2 | ND^*^ | 1.7±0.1 | 32.2 ±0.0 |
| Buffer-N1 | — | — | — | 2.9±0.1 | 2.1±0.0 |

Note: ^*^ means the concentration was not detectable.


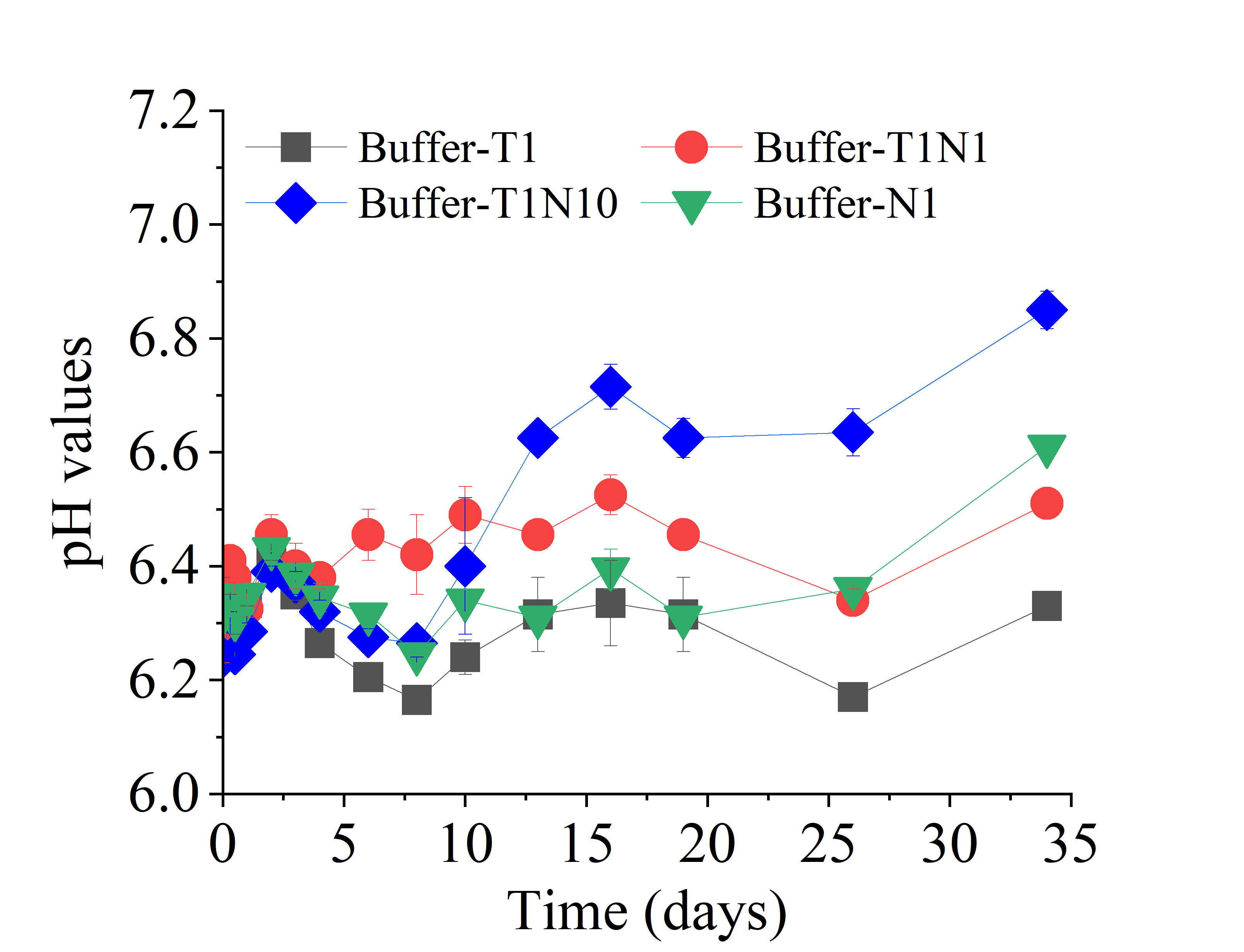


Figure S1. pH variation in the mixed dechlorinating culture with buffering agents.


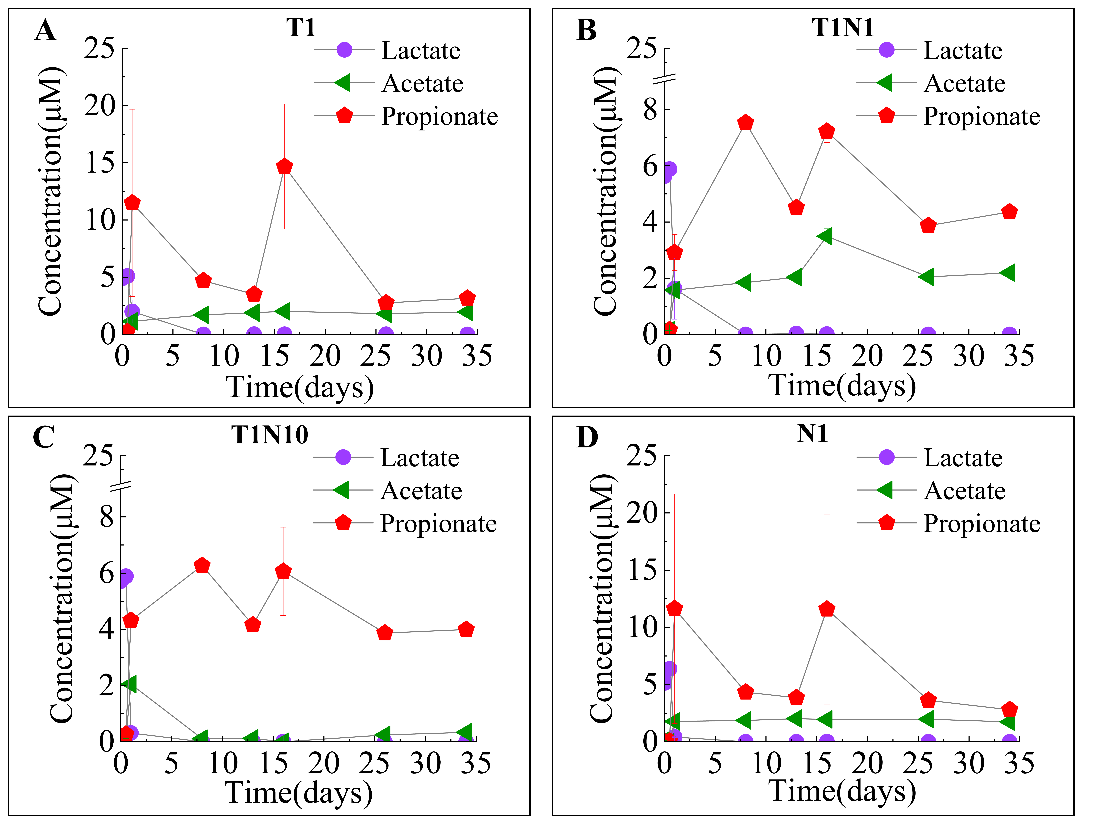


Figure S2. The time course of organic acids concentrations in the TCE- dechlorinating cultures. T1 (A) means the culture added with TCE as sole electron acceptor, the concentration ratio of TCE and nitrate was 1:1 in the culture T1N1 (B), whereas 1:10 in the culture T1N10 (C). and N1 (D) means the culture added with nitrate as sole acceptor.
